# Design and Implementation of a UAV-Based Airborne Computing Platform for Computer Vision and Machine Learning Applications

**DOI:** 10.3390/s22052049

**Published:** 2022-03-06

**Authors:** Athanasios Douklias, Lazaros Karagiannidis, Fay Misichroni, Angelos Amditis

**Affiliations:** Institute of Communication and Computer Systems, National Technical University of Athens, 157 73 Athens, Greece; lkaragiannidis@iccs.gr (L.K.); fay.misichroni@iccs.gr (F.M.); a.amditis@iccs.gr (A.A.)

**Keywords:** UAV, onboard processing, airborne computing, machine learning, image processing, embedded system, testbed

## Abstract

Visual sensing of the environment is crucial for flying an unmanned aerial vehicle (UAV) and is a centerpiece of many related applications. The ability to run computer vision and machine learning algorithms onboard an unmanned aerial system (UAS) is becoming more of a necessity in an effort to alleviate the communication burden of high-resolution video streaming, to provide flying aids, such as obstacle avoidance and automated landing, and to create autonomous machines. Thus, there is a growing interest on the part of many researchers in developing and validating solutions that are suitable for deployment on a UAV system by following the general trend of edge processing and airborne computing, which transforms UAVs from moving sensors into intelligent nodes that are capable of local processing. In this paper, we present, in a rigorous way, the design and implementation of a 12.85 kg UAV system equipped with the necessary computational power and sensors to serve as a testbed for image processing and machine learning applications, explain the rationale behind our decisions, highlight selected implementation details, and showcase the usefulness of our system by providing an example of how a sample computer vision application can be deployed on our platform.

## 1. Introduction

UAVs can either be operated as remotely piloted vehicles or can be pre-programmed to conduct their flight without intervention. Most commercial off-the-shelf (COTS) UAVs almost always include a camera for manual control; there is, however, an increasing degree of autonomous operation capability in the form of return-to-home functions, predefined flight plan execution, etc. These are augmented with an ever more sophisticated obstacle avoidance functionality that exploits various sensors, such as sonars, lidars, and cameras. As an example, DJI’s high-end UAV, Matrice 300 RTK [1], carries 13 cameras, one for remote piloting and 12 for obstacle avoidance, without taking into account any more that are added as a payload. In addition, a great many UAV applications, such as crop monitoring, search-and-rescue (SAR) operations, 3D reconstruction, and visual inspection, rely on the use of dedicated cameras that are fitted onto the UAV in combination with a gimbal for image stabilization and camera-pointing functionality. The extensive use of cameras with UAVs is not without reason. Cameras are excellent sensors for environmental perception, while their attachment to a flying platform and the subsequent increase in the unobstructed field of view (FoV) multiplies their effectiveness. Furthermore, applications that combine computer vision, machine learning, and UAVs, such as orchard management or parcel delivery, now seem plausible thanks to advances in related algorithms [2,3].

To extract useful information from video streams and images, they have to be processed. In recent years, there has been considerable progress in object detection, tracking, and pattern recognition. While processing requirements vary between different methods, there is a clear trend in which more complex and computationally demanding models yield better results. This processing can take place either on the UAV or in a ground station. Processing the sensor’s output on the ground provides access to the latest and most capable processing hardware, but has its shortcomings. Cameras are sensors with considerable bandwidth requirements. A video stream of 30 frames per second and 1.280 × 720 pixels (HD) produces 621 Mbps of data. Transmission of this kind of data stream is only possible using video compression. Video compression introduces latency and a need for processing, and in case the video is to be further processed by computer vision algorithms, both video encoding and decoding are required, thus doubling the latency and processing requirements. Furthermore, transmitting video streams to a ground station makes the UAV dependent on the wireless communication link, thus limiting its operational range, making it susceptible to interference, and bounding its operation to the ground control station. A great deal of research is being carried out to increase the autonomy of UAVs with the goal of realizing completely autonomous machines. While this is not yet the case, many results have already begun to find their way into existing UAVs. Onboard processing is pivotal in this effort.

Designing and building UAV platforms capable of local processing is not without challenges. Processing devices add weight, increase power consumption, and lead to a more complex and expensive UAV system. Moreover, there is a need for extensive hardware and software integration between the sensors, the UAV flight controller, and the companion computer in order to produce a usable system. Today, many researchers build custom UAV systems with various degrees of processing capability and types of sensors to conduct their research. Automated landing of a UAV on a carefully constructed target was achieved by utilizing pattern recognition and local processing in [4,5]. Tournier et al. [6] used a downward-facing camera to estimate the position of a UAV, and a method of extracting the altitude of a UAV from images was presented in [7]. Stereo images were used in [8] to steer a UAV clear of obstacles in its flight path. A customized UAV for SAR operations was proposed in [9], and in [10] a UAV was used for structural health monitoring and concrete crack identification. In [11], General Visual Inspection of aircrafts was attempted, and in [12], a UAV was made to autonomously follow power lines. Finally, the authors of [13] presented a UAV system for capturing hyperspectral images and processing them locally.

The degree of detail about the UAV systems presented in the above publications varies greatly. Some include just an image of the UAV used, while others list the installed sensors, how they were connected, and what the specifications of the individual components are. Most used a single-board computer, such as the Raspberry PI 3 Model B [14], without the necessary processing power to run advanced computer vision algorithms, and they were able to utilize just one camera. The effect of the modifications done to the UAV on maximum flight time was not addressed. More importantly, those UAVs were built for a specific use case and were not intended to serve as a testbed for broad applications. The many different systems built serve as a testimonial for the need for a UAV testbed for computer vision and machine learning applications. Testbeds are highly valued in academia not only because they allow researchers to focus on the innovation aspect of their work, but also because they provide insight into the workings of a system and permit experimentation.

UAVs and their applications have attracted considerable interest from the research community, and as a result, there are many publications describing UAV testbeds with various scopes, such as flight control methodologies [15], path planning [16], or UAV-related wireless communications [17]. However, the published material [18,19,20,21] covering computer vision and machine learning UAV testbeds has several shortcomings. The embedded parallel processing market is dominated by NVIDIA’s Jetson series graphics processing units (GPUs), and the systems presented in the bibliography do not exploit the latest and more powerful embedded GPU from NVIDIA, AGX Xavier, but rather previous or less capable models. The processing unit installed on the UAV plays a crucial role in determining the platform’s competence when it comes to executing the latest and most demanding algorithms. Furthermore, the number and type of cameras are lacking. These two observations are common with the custom-built systems discussed earlier, despite the fact that the latter systems are more advanced. The reason for these limitations comes from the fact that a complex and expensive UAV system is needed to accommodate the aforementioned payloads.

In this article, we present our UAV platform, which has so far been used in one EU-funded research project, ANDROMEDA [22], which focused on cross-border collaboration and information exchange, and it is being utilized in another project, EFFECTOR [23], which develops an interoperability framework for maritime surveillance. To the best of our knowledge, the UAV system presented here is the most complex and up-to-date system in the literature available today. This work attempts to overcome existing barriers and limitations by detailing the architecture and technical aspects of a UAV system designed specifically as a testbed for onboard image processing and machine learning applications. Through the thorough presentation not only of the technical aspects of the system, but also the key requirements that a UAS testbed should meet, we hope to provide researchers with the ability to supplement the system with new technologies and to add more innovation to the system itself.

The rest of the paper is organized as follows. In Section 2, the overall architecture and implementation of the system are presented. This is a large section covering the design of the system and the rationale behind it in detail and providing implementation details about various aspects of the system. Next, in Section 3, the results of the field tests of our platform are described and its capabilities are presented, and in Section 4, an example of the deployment of a sample application is given to show how the platform can be used. Finally, in Section 5, we conclude our article.

## 2. System Design and Implementation

We divide our system into two logical parts: the flight controller with its peripherals (motors, IMUs, GNSS module, etc.) and the companion computer with the cameras that it utilizes. The first part makes up a ready-to-fly system, while the latter provides the capability for onboard processing and visual sensing. The presentation of the system is carried out in sections, following a requirement–solution approach. Key requirements are stated, accompanied by a description of how they are addressed by our design, as well as an explanation of their significance when needed.

### 2.1. Modular, Open-Source, and Easy-to-Use UAV

**Figure 1:**
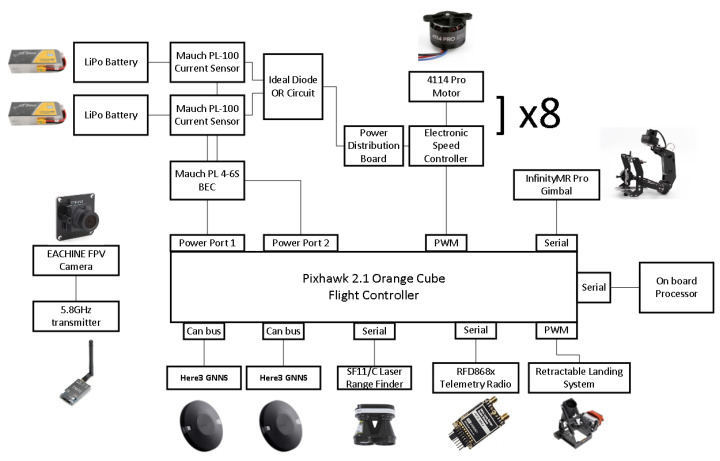
Block diagram of the flight controller and its peripherals.

The type of the UAV—fixed wing or multirotor—is the first major decision to be made. A multirotor-type UAV was selected owing to the ease of use offered by the hover capabilities and the greater payload-lifting capacity for its size in comparison to fixed-wing UAVs [24,25]. Thus, for the UAV frame, we acquired the frame of the DJI Spreading Wings S1000 UAV [26]. It supports an octocopter configuration, is made of carbon fiber with a diagonal wheelbase of 1045 mm, and features retractable landing gear. Next is the flight controller. It should be reliable, allow for interconnection with a companion computer, and be highly customizable. Dependency on a specific manufacturer is not desirable, nor are vendor lock practices that force users to utilize only whatever peripherals are available from the manufacturer. Availability of open-source firmware ensures the former and, in addition, means that there is transparency on how the controller operates. To manage the control of the UAV, we used the pixhawk 2.1 cube orange flight controller [27] while running ArduCopter [28]. Arducopter is an open-source flight stack for multirotor UAVs with a large user base and an ecosystem of compatible hardware modules. Communication with the flight controller is done using the MAVLink protocol, and there is a software-in-the-loop (SITL) simulator available that greatly aids in uploading the software. Due to the existence of multiple APIs [29,30] for interfacing with Arducopter, it is straightforward to connect it to companion computers. Finally, there are more than five types of Arducopter-compatible ground control software (Mission Planner, APM Planner 2, MAVProxy, QGroundControl, UgCS, and others) to choose from. We selected Mission Planner because it is open source with lots of features and it is customizable. Figure 1 presents the block diagram of the flight controller and its peripherals. A detailed component list can be found in Table A3 in Appendix A.

### 2.2. Standalone Remote Piloting System

The purpose of our UAV is to be used for research; thus, the system design is expected to be resilient to erroneous behavior of the application software deployed on board. To ensure that the UAV remains controllable regardless of the state of the companion computer, the two share only a serial connection over which data and commands are exchanged between them. The pilot of the UAV has the option to enable or disable the acceptance of commands sent by the companion computer by switching the flight controller to a mode that disregards anything but the commands coming from the ground control station. In addition, there is a dedicated low-data-rate sub-GHz (868 MHz) radio for the telemetry link, another radio operating at 5 GHz for the first-person view (FPV) video needed by the pilot to fly the UAV, and a third (WiFi 802.11ac) for data transmission and video streaming between the ground station and the companion computer used. Therefore, it is not possible for the processor installed on the UAV to interfere with communication between the UAV and its pilot by generating excessive data traffic on an otherwise shared wireless link.

### 2.3. Airworthy UAV That Can Be Relied upon Even in Cases of Hardware Failure

Since the UAV’s total weight was 12.85 kg, the safety of flying over people and property was a significant concern. Moreover, a UAV system like the one we built is a valuable asset due to the cost, effort, and time spent putting it together. Consequently, we took several steps to increase the system’s resilience, in addition to the low battery and loss of communication fail-safes that were available with the flight controller’s firmware. Choosing a frame with eight motors made it possible to have the UAV continue hovering in case of a motor failure. This is because only 12.5% of the total thrust would be lost, which would be compensated by the rest of the motors. The flight controller utilized two global navigation satellite system (GNSS) receivers, “blending” their outputs. This provided increased position accuracy and enabled the flight controller to continue its operation in case of a GPS glitch by using the output of the unaffected GNSS unit. Moreover, the selected GNSS receivers had a 3D magnetic compass installed, providing further redundancy when it came to calculating the absolute heading of the UAV.

**Figure 2:**
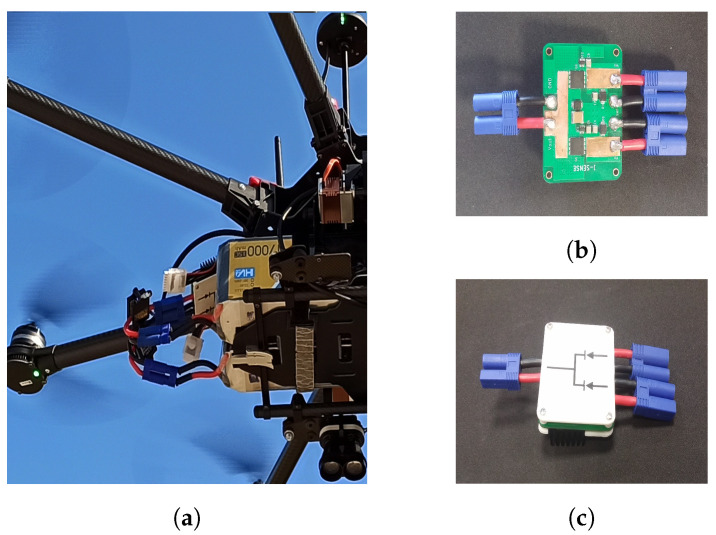
(**a**) The power supply system of the UAV up close. (**b**) PCB view of the ideal diode OR circuit. Notice the copper bars used due to the large currents involved. (**c**) Assembled module with cover and heat sinks.

**Figure A1:**
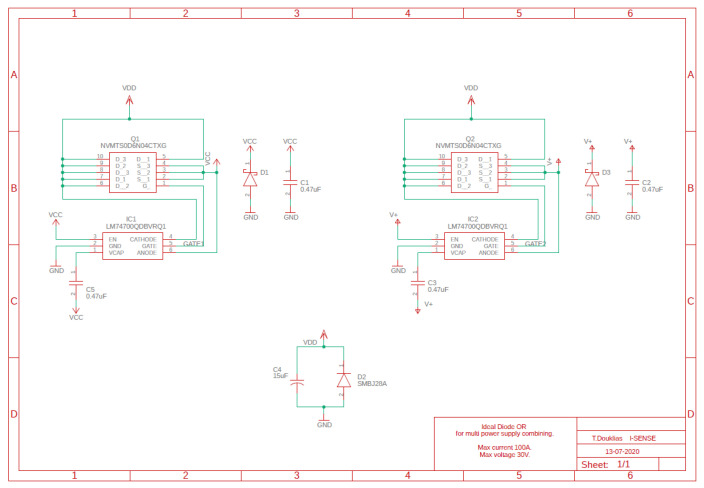
The schematic of the ideal diode OR circuit.

The power supply system consisted of two separate batteries, which were monitored individually and were ORed together to power the UAV. Figure 2 presents the power supply configuration used. Two batteries can be seen to be installed and joined together. Connecting the batteries in parallel is not a good practice. It causes a current spike when they are first connected because of small voltage differences after charging and negates the redundancy coming from having two separate batteries. An option is to use diodes to join the batteries together. This would allow the current to flow only out of the batteries and into the motors and the rest of the UAV’s hardware, but due to the large supply current, around 90 amperes, there would be the need for heat abduction and a sizable heat sink. We thus designed a circuit that behaved as two ideal diodes [31] with their cathodes joined using MOSFET transistors that had the necessary efficiency in handling the supply current with very small heat sinks. The schematic of the circuit is available in Figure A1 in Appendix B, and pictures of it can be seen in Figure 2b,c. An added benefit of this configuration is that batteries of different capacities can be used together.

### 2.4. Ability to Host Various Cameras and to Control and Know Their Orientation

For the system to address multiple use cases, there is a need for different types of cameras or combinations of them to be carried on board. The motors of the UAV cause vibrations, and the UAV’s attitude changes during flight, making it necessary to provide for camera stabilization. In addition, manipulation of the camera’s orientation is required in many applications, such as search and rescue or 3D reconstruction, while knowledge of the camera’s viewpoint provides important metadata for the images and videos produced. The ability for 360-degree unlimited camera rotation in the pan axis decouples the camera’s viewpoint entirely from the UAV’s frame orientation and adds significantly to the platform’s capability. Therefore, a high-quality gimbal is a necessity.

**Figure 3:**
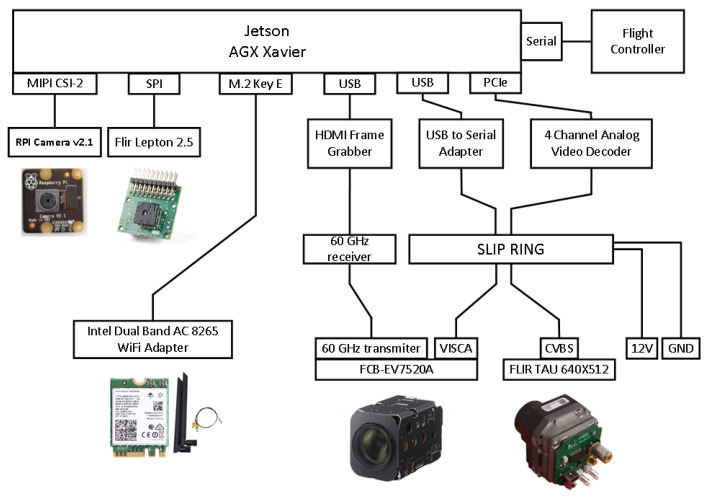
Block diagram of the companion computer and its peripherals.

On the market, there are available gimbals that can receive an arbitrary payload and gimbals integrated with their respective cameras in a ready-to-use solution. However, we found it difficult to acquire a gimbal that could handle more than 1 kg of payload weight, provide three-axis stabilization with 360-degree unlimited rotation in the pan axis, and offer the necessary connections for power and data exchange with its payload. The last requirement is related to the need for a slip-ring connector [32] to be included in the gimbal and to offer the required number and quality of channels for our purposes. The gimbal selected had an 18-channel slip-ring connector. The slip-ring channels were used to pass power and commands to the cameras, as well as to stream analog video back to the processor installed on the UAV. The slip ring’s quality was not sufficient to handle high-definition digital video transmission due to the requirement for high-bandwidth connections. To get around this, we decided to use a wireless video transmission system at 60 GHz with an HDMI input/output interface. The video was transmitted uncompressed, leading to a negligible increase in the video stream latency. The HDMI video output was fed to a USB frame grabber connected to the companion computer for further processing. That way, 360-degree unlimited rotation capability was preserved and various cameras could be fitted as a payload because adaptors for many common camera output interfaces (SDI, component video, LVDS) to HDMI can be readily acquired from the market. The overall arrangement is depicted in Figure 3.

### 2.5. State-of-the-Art Processing Unit Capable of Receiving Multiple Video Streams

**Figure 4:**
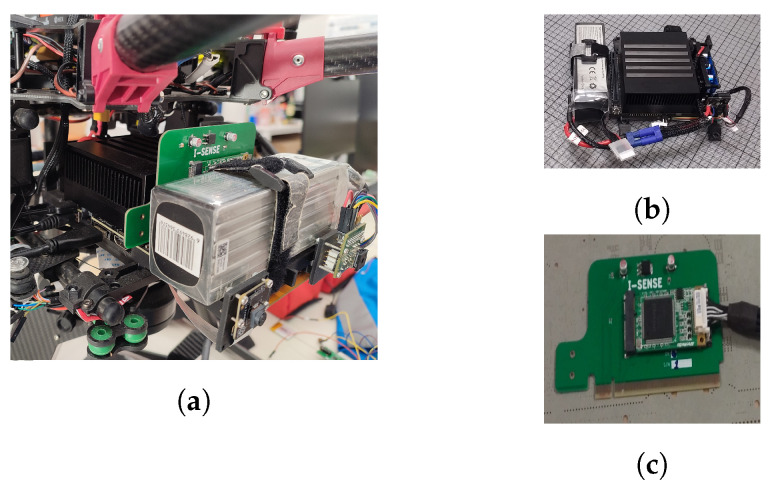
(**a**) The onboard processing system installed on the UAV. The two front-facing cameras can be seen on the bottom right.(**b**) Jetson AGX Xavier (center) with its battery (left) and power supply module (right). (**c**) Custom PCB adapter connecting the analog video decoder to the PCIe bus of the onboard processor.

Many researchers have performed benchmarking of the performance of various embedded devices that can be hosted by a UAV either explicitly [33,34,35,36,37] or implicitly [18,38,39] as part of their work. NVIDIA’s Jetson AGX Xavier embedded processing system [40] is regarded as one of the best choices when it comes to applications that can benefit from hardware support for parallel processing, such as in computer vision or machine learning. It features an eight-core NVIDIA Carmel Armv8.2 64-bit CPU, 512 CUDA cores, 64 Tensor cores, and 32 GB of RAM. In addition, it offers many connectivity options. It has three USB ports, SPI, and UART buses, an 8x PCIe port, and can host up to six MIPI CSI cameras. The SPI bus is taken up by a front-facing Flir Lepton 2.5 thermal camera. Next to it, there is an RGB Raspberry Pi camera v2.1 connected to one of the MIPI CSI slots. One USB port is taken up by the HDMI to the USB frame grabber, while the PCIe port hosts a four-channel analog video decoder that receives the output of a Flir TAU 2 thermal camera. Due to the restricted space available, we were forced to select a small-form-factor device with a mini-PCIe interface and to design a custom PCB adapter (Figure 4) to connect the mini-PCIe board to the PCIe slot of the companion computer. All in all, there were four cameras providing video streams with the capability for 10 more.

### 2.6. Powerful Cameras for the Applications

**Figure 5:**
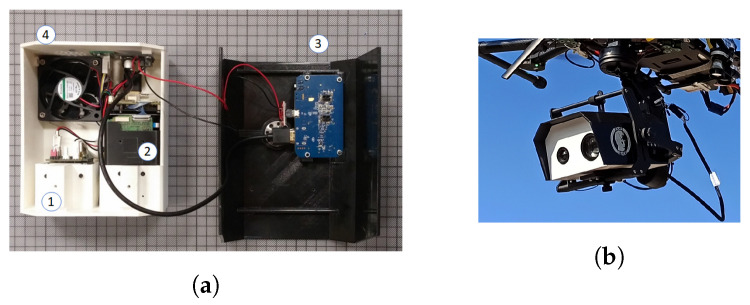
(**a**) The interior of the camera unit. (1) Thermal camera, (2) RGB camera, (3) HDMI wireless transmitter, (4) fan for heat abduction, and (**b**) gimbal with the camera unit under the UAV.

The cameras installed on the UAV fell into two broad categories. There were those used for sensing of the surrounding environment with the purpose of supporting autonomous operation or piloting aids, which were usually of a low cost, without stabilization, and more numerous, and those used for the specific use case. They could be RGB cameras with optical zoom capability or hyperspectral or thermal cameras depending on the application, and they were generally state-of-the-art sensors for their respective types with considerable cost and weight. As stated earlier, many different types and combinations of cameras can be integrated into our platform. Here, we provide an example of the integration of a thermal and optical zoom-capable RGB camera. We designed and 3D-printed a case that hosted the two cameras, along with their power supply modules and the HDMI video transmitter, in a single unit that was, in turn, secured onto the gimbal. Details can be seen in Figure 5.

### 2.7. Abstraction Layer for Easy Access to the Flight Controller

The key objective of our platform was to abstract away the specific details of the UAV implementation in order to allow for the researchers to focus on their algorithms. To achieve this, we developed an abstraction layer, an API with its backend daemon, that enabled retrieval of data from the autopilot and transmission of commands in a uniform and easy-to-understand way. Splitting the application software from the software that handled the communication with the flight controller led to the well-known producer–consumer problem, since the two needed to exchange data, which were either flight information or commands. To solve this problem, we utilized the shared file memory mapping interprocess communication mechanism (IPC). We did so for the following reasons:

- The concept of exchanging data and issuing commands by writing to or reading from memory locations leads to an intuitive solution that requires little time to grasp. Each piece of information is associated with a file with a relevant filename, such as the UAV’s latitude with a file named lat in folder gnss. Thus, the filenames of the files and their organization in directories make it straightforward to choose the file that contains the intended information.
- Shared memory mappings can be created by invoking the portable operating system interface (POSIX) compliant mmap [41] system call. Since it is possible to use system calls in many programming languages, the same is true for our abstraction layer; it can be used by any programming language that supports using system calls.
- The files used for interprocess communication can be mapped by multiple programs, and each program can use only the files that are of interest to it. This makes it easy to split the application software into several processes, either to serve a certain architectural choice or for division of work between the members of a team.
- Process synchronization can be done by using file locks on the memory-mapped files. Similarly to mmap, there is the fcntl [42] system call, which can be used to lock access to a file. In addition, there is the possibility for fine-grained synchronization through the use of exclusive (write) or shared (read) locks.
- Our goal is not only to provide the most up-to-date information about the UAV, but also to make it always available, and that means that the data written to the files should not be removed when they are read. The persistence provided by memory-mapped files compared to other IPC mechanisms, such as message queues or sockets, fits well with our use case.

**Figure 6:**
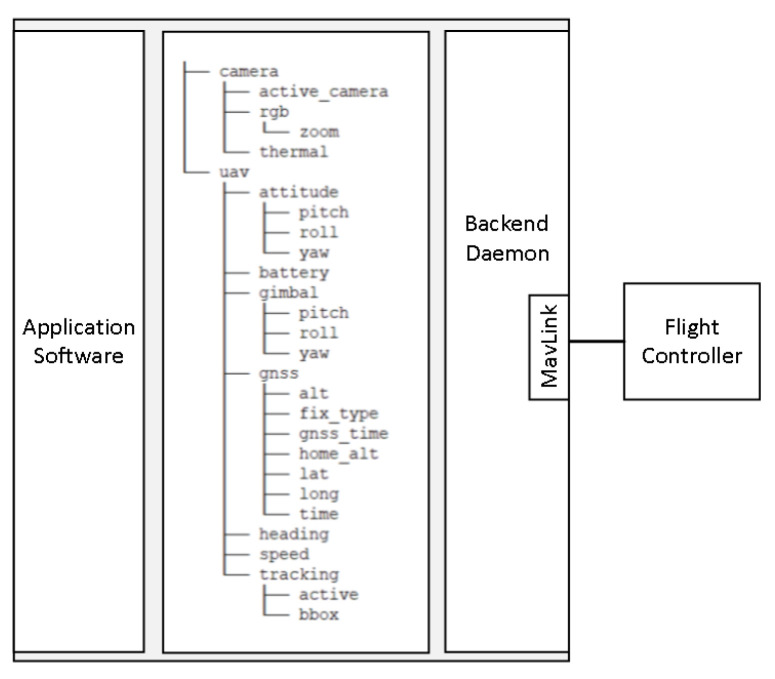
Architecture of the software abstraction layer for communication with the flight controller.

In Figure 6, the overall architecture of the software abstraction layer is presented. The backend daemon retrieves data from the flight controller using the MAVLink protocol at a constant frequency and, consequently, writes this information to the corresponding files to be read by the application software.

For sending commands, the contents of the corresponding files are transformed into MAVLink commands and sent to the flight controller at regular intervals. The repeated issuing of commands to the flight controller does not create a problem because, once the command is executed, the same command has no effect. For example, commanding the UAV to switch to an absolute yaw of 0 degrees (face North) does not produce any change in the UAV’s attitude once the corresponding orientation has been achieved.

### 2.8. Flight Time

**Figure 7:**
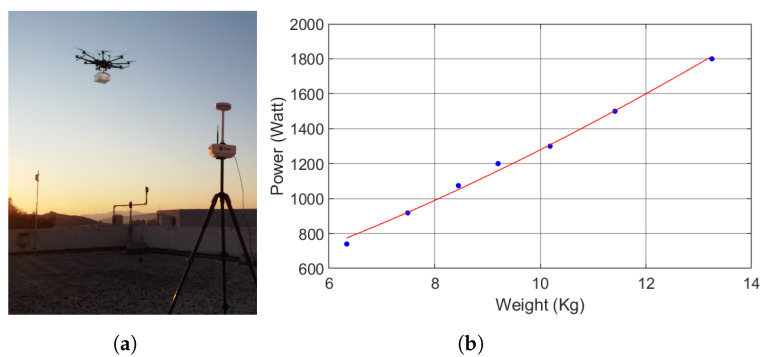
(**a**) The UAV with a box attached underneath for power consumption vs. weight measurement. (**b**) Equation (2) plotted against the power–weight measurements, which can be found in Table A1 in Appendix A.

Flight time is a key specification of UAVs and is highly sought after by end users. In our case, the flight time must be sufficient to execute various scenarios for the evaluation of the application software deployed on board. We targeted a flight time between 15 and 20 minutes at 80% of the battery capacity, which is consistent with the performance of most COTS UAVs [25]. The flight time is estimated by the ratio of the available energy to the consumed power when hovering. The consumed power is a function of the UAV’s weight, and the available energy depends on the batteries installed. We derived an expression of the flight time with respect to the battery weight to be able to make a decision about the needed battery weight and expected flight time. Using momentum theory, the power consumption with respect to the weight of the UAV can be found to be [43]:

(1) $$P\left(m\right)=\frac{{\left(mg\right)}^{3/2}}{FM\;\eta_{motor}r_{p}\sqrt{2N_{r}\rho \pi }}$$

where *m* is the UAV’s total weight (in kg), *g* is the acceleration due to gravity, FM is the figure of merit of the propellers, $\eta_{motor}$ is the motor efficiency, $r_{p}$ is the blade radius, $N_{r}$ is the number of rotors, ρ is the density of the air, and *P* is the consumed power in watts. Because the manufacturer of the motors does not make available the necessary specifications to apply Equation (1), we collected measurements of power consumption versus weight in order to estimate the power consumption. To this end, we attached a plastic box under the UAV and progressively loaded it with increasing weight to measure the respective power consumption. Then, a relationship of the form $P=a\cdot m^{3/2}+b$ was fitted to the measurements with the LSE method. Parameter *b* accounts for offsets in the power and weight measurements, as well as power consumption not related to flying. The curve fitted to the measurements is depicted in Figure 7, along with a picture of the measurement procedure. The end result is:

(2) $$P\left(w\right)=32.2\cdot m^{3/2}+260$$

**Figure 8:**
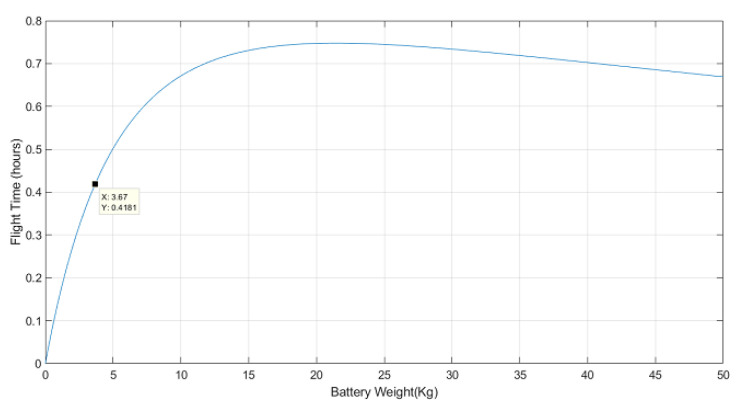
Flight time of the UAV with respect to the battery weight installed.

Lithium–polymer (LiPo) batteries are the dominant battery type used to power UAVs due to their high energy-to-weight ratio, discharge capability, life cycle, and cost [44,45]. Their energy capacity has been found to be linear with respect to their mass [46]. The specific energy of the LiPo batteries available on the market is around 0.2 Wh/g (see Table A2 in Appendix A for more details). Since the UAV weighs 9.25 kg without any batteries installed, the flight time in hours is:

(3) $$T\left(w_{bat}\right)=\frac{E(w_{bat})}{P(9.25+w_{bat})}=\frac{0.2\cdot 10^{3}\cdot w_{bat}}{32.2\cdot {\left(9.25+w_{bat}\right)}^{3/2}+260}$$

with $w_{bat}$ being the battery weight in kg. To ensure a flight time of at least 15 minutes at 80% battery capacity, a pair of batteries with a total weight greater than 2.4 kg is required, according to Equation (3). Commercially available LiPo batteries come at manufacturer-specific capacities and weights, leading to a finite number of possible weight choices. Another important consideration is the fact that the batteries account for the single heaviest component on the UAV, and their weight and placement plays a crucial role in having a well-balanced platform. Therefore, we made the decision not to increase the weight of the batteries any further once a good weight distribution had been achieved. Two 6 S 17,000 mAh LiPo batteries, with a total battery weight of 3.67 kg, were selected. They should provide for 19 minutes of flight time at 80% of the battery capacity. Figure 8 shows the expected flight time with respect to the weight of the batteries installed on the UAV and the selected battery weight.

## 3. Field Test Results

**Figure 9:**
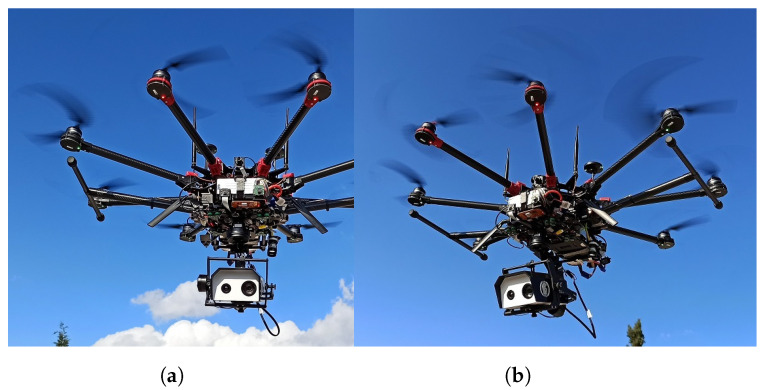
(**a**) Front view of the UAV while flying. (**b**) Side view of the UAV.

Figure 9 shows the UAV airborne during field testing. The stabilized cameras underneath the UAV stand out as the most noticeable hardware component. The rest of the equipment is packed between the rotor plane and the gimbal. The landing gear can be seen to be retracted to allow for an unobstructed 360-degree field of view. The two vertical antennas on the front top side of the UAV are for the sub-GHz telemetry link. The WiFi utilizes a set of antennas placed on each side of the UAV with a 45-degree inclination, while the disc-shaped objects on the back of the UAV’s top side are the GNSS antennas.

**Figure 10:**
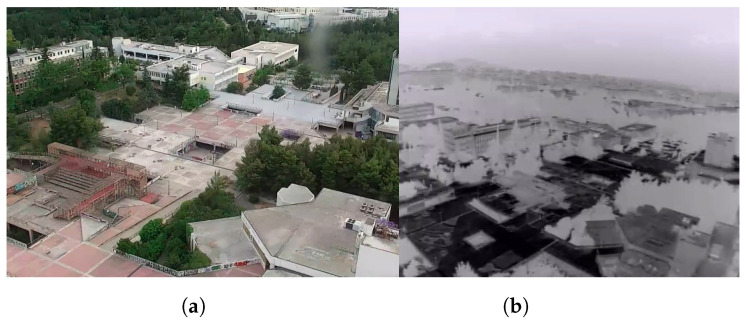
(**a**) View of the university campus from the RGB camera onboard the UAV. (**b**) View of the same area with the thermal camera. The thermal camera’s color space is black (hot).

We spent many hours testing our system and ensuring the correct operation of the developed software. During the tests, with the entirety of the system working, we verified that the flight time at 80% of the battery capacity was 18 minutes, which was close to the calculated value. The total power consumed by the system when loitering was approximately 2000 watts. The thrust variation between the motors when loitering was less than 5%, which indicated a good overall distribution of the weight on the UAV. In addition, we explored the capabilities of the sensors installed on the UAV. In Figure 10, the UAV flew over the campus of the NTUA University in Athens and captured pictures using the RGB and thermal camera.

**Figure 11:**
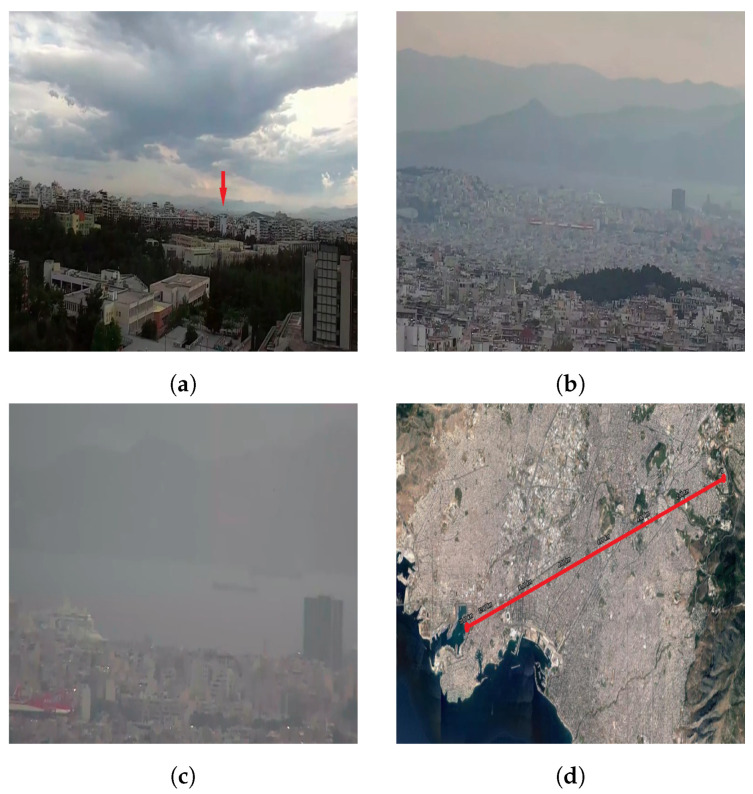
(**a**) View from the RGB camera without any magnification. Red arrow marks the point of observation. (**b**) Ten-times magnification. (**c**) Thirty-times magnification. (**d**) The distance between the UAV and the point of observation (13 km) plotted on a map. The UAV is located at the top right end of the red line and the port at the bottom left line’s end.

To test the zoom of the RGB camera, we attempted to get a view of the port of Piraeus from the university campus, a distance of approximately 13 km. In Figure 11, shots of the port at different zoom levels can be seen, along with the point of observation (red arrow) and the UAV’s position on the map. Without any magnification, the sea can barely be recognized in the background of Figure 11a. At a magnification of 10 times (Figure 11b), a cruise ship docked at the harbor can be seen. Other ships sailing through the port can be identified with great difficulty. Finally, in Figure 11c, the maximum possible magnification is employed (30×). Aside from the docked cruise ship, two more ships can be seen. By comparing Figure 11a with Figure 11c, it becomes evident that the RGB camera is able to reveal objects at great distances. This capability is only possible through the UAV–camera combination. No matter how powerful a camera is, without the obstacle clearance provided by the UAV, such long-range observation capabilities would not be feasible.

**Figure 12:**
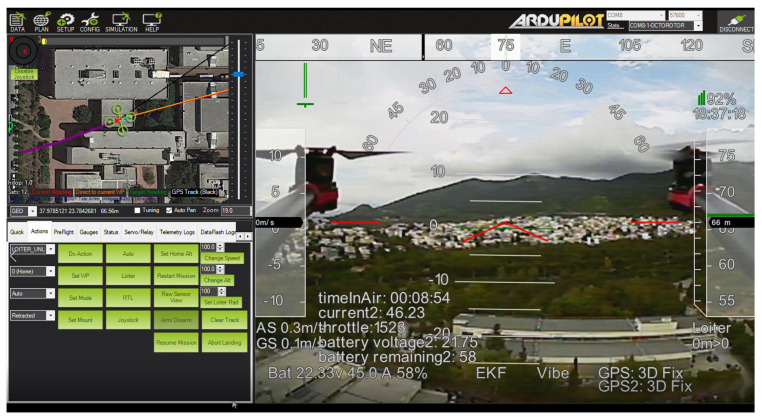
Mission Planner’s user interface as it was configured during the flight tests.

A screenshot of the Mission Planner’s user interface is presented in Figure 12. There are three main panels: a map with the UAV’s position, several action buttons grouped into tabs, and an FPV video feed. This is a common layout for ground control software. The FPV video is overlayed with various information important to the pilot, such as the time in the air, current consumption and battery remaining, GPS status, altitude, etc.

## 4. Deployment of a Sample Application on the UAV

In addition to the flying and sensing capabilities presented previously, easy deployment of new software is crucial for our platform to fulfill its role as a testbed. In this section, we present how a computer vision application that tracks an object with a camera can be deployed on the UAV.

The tracking algorithm employed is DeepSORT [47]. DeepSORT is a tracking-by-detection algorithm. Through the use of an object detector, it processes each frame by looking for objects and tries to associate them with detections performed in the previous frames using spatial distance and visual information. The usage of an object detector by DeepSORT enables the algorithm to output information about the type of tracked object and makes it possible for the tracking procedure to begin without the need for an initial designation of the objects to be tracked. The deployed implementation of DeepSORT uses the Yolov4 [48] convolutional neural network (CNN) for object detection. Yolov4 is a one-stage object detection model with state-of-the-art inference speed and average precision combination. In addition, there are freely available weight sets produced by training the model on a variety of datasets, which makes it readily applicable in a wide range of applications.

**Figure 13:**
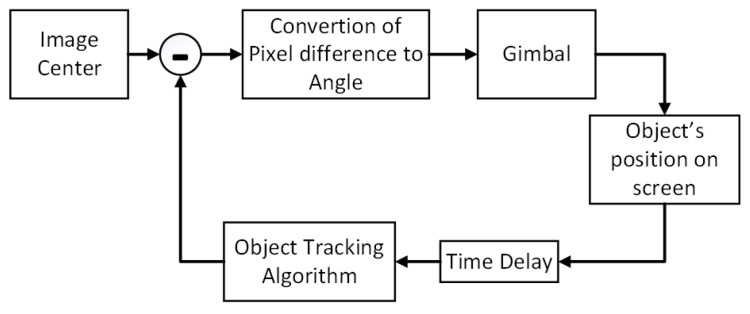
Block diagram of the sample tracking application.

The block diagram of the sample application is presented in Figure 13. Once an object has been detected, tracking begins; each frame is processed, and a bounding box that includes the object, a label, and an ID number is computed. The pixel difference of the box’s center with the center of the image is computed and converted into an angle using the camera’s FoV angle with the following formula:

(4) $$Angle=\frac{Distance\_from\_the\_center\_of\_the\_image\_in\_pixels}{Image\_size\_in\_pixels}\cdot FoV$$

The computed value is subsequently written to the corresponding memory-mapped file to signal the gimbal to turn the camera. This process is repeated with a fixed time delay to allow for the camera to turn before a new correction for the camera’s orientation is computed. In case there are multiple objects detected, only the first detected object is taken into account, and the rest are ignored.

The end result is that the camera turns to keep the tracked object at the center of every frame. It is important to note that:

- OpenCV is used to grab frames from the camera and feed them into the tracker. The camera can be accessed in OpenCV as if it were a standard USB camera connected to a PC running Linux.
- Instructing the gimbal to turn the camera is straightforward. Once the new angle is computed, it just has to be written to the correct file.
- Initiating the tracking procedure and monitoring the results does not require the development of any additional software. Thanks to having a full-stack Linux PC with WiFi connection, a remote desktop application can be used to gain access to a graphical user interface environment.

**Figure 14:**
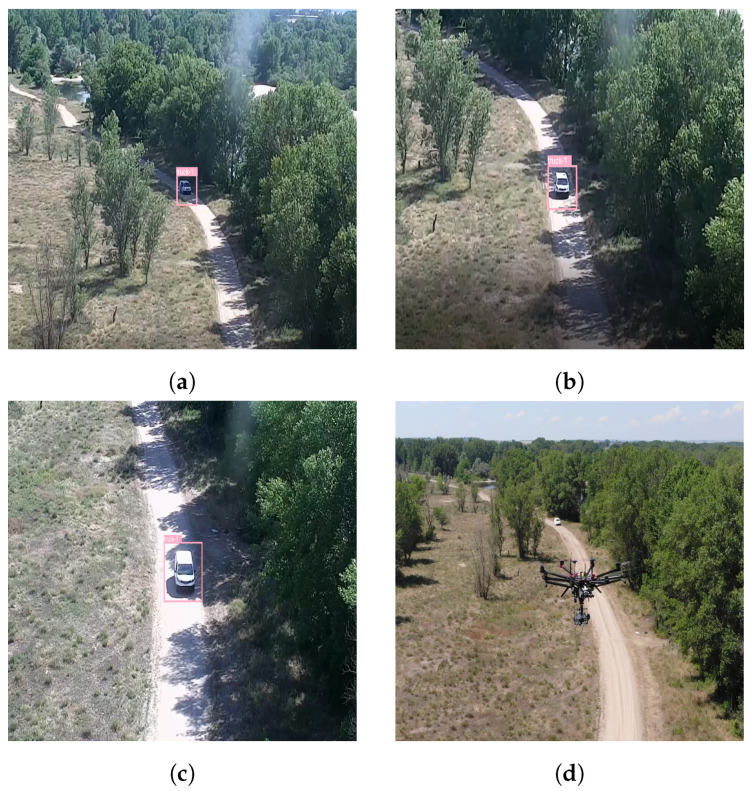
(**a**–**c**) Tracked vehicle marked with the pink bounding box as it approaches the UAV. (**d**) The UAV flying with the tracked vehicle in the background.

Consequently, there is little difference in running the application on a researcher’s PC and the onboard processor. Figure 14 includes several screenshots from the tracking of a vehicle using the aforementioned sample application. The vehicle is detected by the Yolov4 CNN, marked with a pink bounding box, and labeled as a truck. Since it is the first object detected, it receives the identification number 1. As the vehicle moves towards, the UAV the camera view can be seen to change to keep the vehicle centered.

## 5. Conclusions

In this paper, we presented the design and implementation of our UAV testbed for computer vision and machine learning applications. Essential requirements for the testbed were listed along with the corresponding hardware or software implementation. The complexity and capabilities of our platform set it apart from other similar testbeds. We went to great lengths to capture the requirements that we believe make it suitable to be used as a testbed and explained how they were met. By doing so, we hope that our experience will provide other researchers with valuable insight into designing their platforms.

The proposed platform was built and tested, and an example of how it can be used to evaluate the performance of a real application was made. Evaluation of a proposed solution to a problem and comparison of the results with other existing solutions are at the heart of the research process. Our system makes it possible to deploy and evaluate solutions without much effort, thus accelerating the evaluation process and freeing time to focus on the problem under investigation and its solution.

An interesting finding is related to the power consumption of the companion computer and its peripherals. When fully utilized, 70 watts of power are required, a significant amount for an embedded device. Nevertheless, given the power needed to keep the UAV airborne, 2000 watts, the power consumed for processing is negligible (less than 4%). Given this, it is evident that even more powerful processing units can be fitted on UAVs because the power consumption limitation can somewhat be relaxed. However, it is essential to note that in addition to supplying power to the companion computer, attention must be paid to heat abduction and the weight of the cooling solution so as not to unnecessarily impact the flight time.

Despite having developed a software abstraction layer that, among other things, enables multiple applications to run concurrently, the resources of the processing unit have to be shared. Consequently, there is a limitation on the number and complexity of the algorithms that can be executed at any given time in parallel. The field of embedded processing devices for parallel computing is under intensive development, and in the future, new and more powerful devices will be available to address this limitation.

In addition to its use as a testbed for computer vision and machine learning applications, our platform has the potential to be used in other fields. We plan to add a 5G modem and a sub-GHz radio to enable the companion computer to become part of a cloud infrastructure to explore distributed computing and the benefits that it can bring in resource-intensive applications, such as 3D reconstruction, object detection, and optical inspection. The sub-GHz radio can be used to make the UAV part of a mesh network, transmit commands to other UAVs, or collect data from sensors on the ground. 

## Data Availability

Not applicable.

## Abbreviations

The following abbreviations are used in this manuscript:

API	Application Programming Interface
COTS	Commercial off the Self
CSI	Camera Serial Interface
FPV	First-Person View
GNSS	Global Navigation Satellite System
IMU	Inertial Measurement Unit
IPC	Interprocess Communication
LVDS	Low-Voltage Differential Signaling
MAVLink	Micro Air Vehicle Link
NTUA	National Technical University of Athens
POSIX	Portable Operating System Interface
SDI	Serial Digital Interface
SPI	Serial Peripheral Interface
UART	Universal Asynchronous Receiver–Transmitter
UAS	Unmanned Aircraft System
UAV	Unmanned Aerial Vehicle

## Appendix A. Miscellaneous Tables

**Table A1:** Power consumption with respect to UAV weight measurements.

Weight (kg)	Voltage (V)	Current (A)	Power (W)	Power Loading (g/W)
6.335	22.6	31	740	8.56
7.490	22.4	41	918	8.16
8.450	21.49	50	1074	7.86
9.200	21.51	54	1200	7.66
10.185	21.47	63	1300	7.83
11.415	21.11	76	1500	7.61
13.250	20	91	1800	7.36

**Table A2:** Specific energy of 6 S 17000 mAh LiPo batteries of various brands according to the data published by their manufacturers.

Manufacturer Brand	Energy Capacity (Wh)	Weight (kg)	Specific Energy (Wh/g)
Etop Power	377.4	1.95	193.5
Maxamps	377.4	1.928	195.7
Overlander	377.4	2.04	185
Tattu	377.4	1.847	204

**Table A3:** Detailed list of the hardware components used and their costs.

Description	Quantity	Weight (kg)	Cost (€)
UAV frame ${}^{1}$	1	DJI Spreading Wings S1000	2450.00
UAV Battery	2	Tattu LiPo 6 S 17,000 mAh 387.6 Wh	337.38
Companion computer Battery	1	Tattu LiPo 6 S 4500 mAh 99.4 Wh	103.67
Telemetry Radio	1	RFD 868+ Modem	245.00
GNSS receiver	2	Hex Here 3—CAN GPS	178.50
Laser Altimeter	1	LightWare SF11/C	301.32
Current Sensor	2	Mauch PL100	35.41
Flight Controller	1	Pixhawk 2.1 orange cube	215.00
Onboard processor	1	Jetson AGX Xavier	710.52
RGB Camera	1	Sony FCB-EV7520A	819.00
Thermal Camera	1	Flir TAU 640	5299.20
FPV Thermal Camera	1	Flir Lepton 2.5	297.60
FPV RGB Camera	1	Raspberry Pi Camera v2.1	52.64
Pilot Video transmission system	1	Eachine ROTG01 Pro UVC OTG 5.8G Pro	35.00
HDMI frame grabber	1	Magewell USB Capture HDMI Gen 2	415.37
Analog video decoder	1	Avermedia C351 (Intersil TW6865)	116.68
Gimbal	1	HDairstudio Infinity MR Pro	1734.00
Total cost			13,897.58

^1^ The motors, propellers, and landing gear are included with the UAV frame.
